# Screening for Obstructive Sleep Apnea Risk by Using Machine Learning Approaches and Anthropometric Features

**DOI:** 10.3390/s22228630

**Published:** 2022-11-09

**Authors:** Cheng-Yu Tsai, Huei-Tyng Huang, Hsueh-Chien Cheng, Jieni Wang, Ping-Jung Duh, Wen-Hua Hsu, Marc Stettler, Yi-Chun Kuan, Yin-Tzu Lin, Chia-Rung Hsu, Kang-Yun Lee, Jiunn-Horng Kang, Dean Wu, Hsin-Chien Lee, Cheng-Jung Wu, Arnab Majumdar, Wen-Te Liu

**Affiliations:** 1 Centre for Transport Studies, Department of Civil and Environmental Engineering, Imperial College London, London SW7 2AZ, UK; 2 Department of Medical Physics and Biomedical Engineering, University College London, London WC1E 6BT, UK; 3 Parasites and Microbes Programme, Wellcome Sanger Institute, Hinxton CB10 1RQ, UK; 4 Chemical Engineering and Biotechnology, University of Cambridge, Cambridge CB3 0AS, UK; 5 Cognitive Neuroscience, Division of Psychology and Language Science, University College London, London WC1H 0AP, UK; 6 School of Respiratory Therapy, College of Medicine, Taipei Medical University, Taipei 110301, Taiwan; 7 Sleep Center, Shuang Ho Hospital, Taipei Medical University, New Taipei City 235041, Taiwan; 8 Department of Neurology, Shuang Ho Hospital, Taipei Medical University, New Taipei City 235041, Taiwan; 9 Department of Neurology, School of Medicine, College of Medicine, Taipei Medical University, Taipei 110301, Taiwan; 10 Taipei Neuroscience Institute, Taipei Medical University, Taipei 110301, Taiwan; 11 Dementia Center, Shuang Ho Hospital, Taipei Medical University, New Taipei City 235041, Taiwan; 12 Department of Medical Imaging and Intervention, Chang Gung Memorial Hospital at Linkou, Taoyuan 33305, Taiwan; 13 Division of Pulmonary Medicine, Department of Internal Medicine, Shuang Ho Hospital, Taipei Medical University, New Taipei City 235041, Taiwan; 14 Department of Physical Medicine and Rehabilitation, Taipei Medical University Hospital, Taipei 110301, Taiwan; 15 Research Center of Artificial Intelligence in Medicine, Taipei Medical University, Taipei 110301, Taiwan; 16 Graduate Institute of Nanomedicine and Medical Engineering, College of Biomedical Engineering, Taipei Medical University, Taipei 110301, Taiwan; 17 Department of Psychiatry, Taipei Medical University Hospital, Taipei 110301, Taiwan; 18 Department of Otolaryngology, Shuang Ho Hospital, Taipei Medical University, New Taipei City 235041, Taiwan

**Keywords:** obstructive sleep apnea, polysomnography, anthropometric features, random forest, visceral fat level

## Abstract

Obstructive sleep apnea (OSA) is a global health concern and is typically diagnosed using in-laboratory polysomnography (PSG). However, PSG is highly time-consuming and labor-intensive. We, therefore, developed machine learning models based on easily accessed anthropometric features to screen for the risk of moderate to severe and severe OSA. We enrolled 3503 patients from Taiwan and determined their PSG parameters and anthropometric features. Subsequently, we compared the mean values among patients with different OSA severity and considered correlations among all participants. We developed models based on the following machine learning approaches: logistic regression, k-nearest neighbors, naïve Bayes, random forest (RF), support vector machine, and XGBoost. Collected data were first independently split into two data sets (training and validation: 80%; testing: 20%). Thereafter, we adopted the model with the highest accuracy in the training and validation stage to predict the testing set. We explored the importance of each feature in the OSA risk screening by calculating the Shapley values of each input variable. The RF model achieved the highest accuracy for moderate to severe (84.74%) and severe (72.61%) OSA. The level of visceral fat was found to be a predominant feature in the risk screening models of OSA with the aforementioned levels of severity. Our machine learning models can be employed to screen for OSA risk in the populations in Taiwan and in those with similar craniofacial structures.

## 1. Introduction

Obstructive sleep apnea (OSA) refers to sleep-disordered breathing caused by partial or complete airway obstruction [1]. This disease has become a global health concern, with approximately one billion people aged 30–65 years being affected by mild-to-severe OSA, and 425 million having moderate to severe OSA [2]. In the United States, the prevalence rate of OSA increased by approximately 30% between 1990 and 2010 [3]. OSA is regarded as a risk factor for various comorbidities, including 2–3-fold increased risks of cardiovascular and metabolic diseases [4], and decreased hippocampal volume, which is associated with neurocognitive deficits [5]. Therefore, early diagnosis of and suitable treatment for OSA are essential.

In-laboratory polysomnography (PSG) is the standard measurement to diagnose OSA and differentiate severity. Specifically, the apnea–hypopnea index (AHI), which records the total number of apnea and hypopnea events during sleep time, is determined using PSG data; the index is used to differentiate between four OSA severity categories: normal (AHI < 5), mild (5 ≤ AHI < 15 events/h), moderate (15 ≤ AHI < 30 events/h), and severe (AHI ≥ 30 events/h) [6]. Curative interventions are generally recommended for patients with moderate or severe OSA (AHI ≥ 15 events/h). Despite its usefulness, PSG has some clinical shortcomings. For example, PSG requires a lengthy monitoring time and the involvement of licensed technicians; thus, the average PSG waiting time in developed countries ranges from months to 2 years [7]. The time-consuming and labor-intensive nature of PSG may limit its efficiency and effectiveness. Alternative methods have been proposed to improve measurement accessibility, including the OSA questionnaires and home sleep tests (HSTs) through oximetry; however, none of them is fully reliable as a surrogate for PSG. A review study indicated that inconsistent results from studies using different OSA questionnaires (Berlin; apnea score; sleep apnea scale of the sleep disorders questionnaire; snoring, tiredness, observed-apnea, and high blood pressure (STOP); STOP including body mass index (BMI), age, neck circumference, and sex) can be attributed to heterogeneity in study design and enrolled populations [8]. Regarding HSTs, despite offering convenient diagnosis, this approach may be insufficiently accurate to rule out OSA when the respiratory events of patients are mainly associated with arousals [9]. Moreover, because of the reduced number of physiological channels in HSTs, this approach may not be suitable for patients with complicated comorbidities [10]. Given the aforementioned deficiencies in current methods, novel models to rapidly screen for OSA risk and thereby increase the efficiency of the therapeutic decision-making process are required.

To develop clinically applicable models, exploring the relationships between OSA severity and anthropometric features may be worthwhile. Sex, age, and BMI for instance, have been suggested as useful indicators in OSA risk screening [11]. In one study, the prevalence of moderate to severe OSA in middle-aged men (aged 30–49 years) and in older men (aged 50–77 years) was 3.3-fold and 1.9-fold higher than the values of female cohorts with the same age ranges, respectively [12]. Another study indicated that those with obesity (BMI: 30–39.9 kg/m^2^) had higher mean AHI and oxygen desaturation index (ODI) values than those with a healthy weight (AHI: 28.5 ± 1.22 events/h vs. 14.3 ± 1.40 events/h; ODI: 32.1 ± 1.20 events/h vs. 15.8 ± 1.40 events/h, all *p* < 0.01) [13]. Neck and waist size have also been adopted as proxies for BMI when screening for OSA risk, with a neck size of >43 cm in men or >38 cm in women and a waist size of >102 cm for both sexes indicating increased risk [14]. In another study, neck size (ρ: 0.54), waist size (ρ: 0.75), and body water (ρ: 0.69) were all significantly and positively correlated with AHI [15]. Moreover, body fat level was significantly correlated with AHI (r = 0.65), and abdominal visceral fat level calculated through cross-sectional computed tomography exhibited adequate sensitivity and specificity (*p* < 0.01) in differentiating between those with OSA and healthy individuals [16]. Hence, these easily acquired anthropometric features may be useful in OSA risk screening models because their associations with AHI have been demonstrated.

In this retrospective study, we sought to develop risk screening models for OSA using machine learning approaches and easily acquired parameters, such as anthropometric features. We hypothesized that anthropometric features (e.g., body profile and body composition parameters), which are associated with OSA severity, would be beneficial in models for screening the risk of moderate to severe OSA (AHI ≥ 15) and severe OSA (AHI ≥ 30). We developed OSA risk screening models using various machine learning approaches that incorporated easily accessed anthropometric features. Subsequently, we compared the means of the obtained anthropometric features in groups with different OSA severity. We also examined the correlations between anthropometric features and sleep quality indices. The aim of these analyses was to elucidate the relationships between these variables.

## 2. Materials and Methods

### 2.1. Ethics

The Ethics Committee of the Taipei Medical University-Joint Institutional Review Board reviewed and approved the protocol of this retrospective study (TMU-JIRB No: N201911007). All relevant procedures for data collection, analysis, and preservation were conducted per the approved protocol.

### 2.2. Study Population

We retrospectively collected the data of patients who underwent PSG for OSA severity assessment at the Sleep Center of Taipei Medical University–Shuang Ho Hospital (New Taipei City, Taiwan) between May 2019 and December 2021. The inclusion criteria for data use were as follows: age between 18 and 90 years, overall PSG recording time of >6 h and sleep efficiency of >60%, no history of invasive surgery for OSA, and no regular use of hypnotic or psychotropic medications. Using the medical registration number list of the eligible individuals, we acquired their physical profiles, which included information on age, sex, body mass index (BMI), and neck and waist circumferences, from their responses to a baseline survey questionnaire recorded in a sleep center database. Next, we obtained data regarding the participants’ medication and surgical history from their clinical records. Because of known correlations between OSA severity and craniofacial features, we collected data from only Han individuals to limit the effect of craniofacial feature disparities [17].

### 2.3. Body Composition

Body composition data were collected from the aforementioned sleep center database. The procedures used for determining body composition are described below. Before the patients underwent PSG, we measured their body compositions (through bioelectrical impedance) using the Tanita MC-780 system (Tanita, Tokyo, Japan). Before the measurement, the patients fasted for 3 h and emptied their bladder. During data reading, the patients were instructed to stand still and hold the detection handles with both arms straight down while ensuring their inner thighs did not touch. Fat mass and fat-free mass (comprising bone and muscle mass) in various body regions (whole body, only limbs, and only trunk) were assessed, and the percentages of fat and muscle in the aforementioned regions were subsequently derived. Visceral fat level (as an index for evaluating fat encompassing the vital organs in the abdominal cavity; range 1–55I), basal metabolic rate (as the minimum energy required by the body at rest), and physique rating (body fat mass divided by muscle mass) were also determined. To evaluate water distribution in the body, the volume of total body water (TBW), including the volumes of extracellular water (ECW) and intracellular water (ICW), percentage of body water, ratio between ECW and ICW, and ratio between trunk fat and whole-body fat were determined. All the derived parameters were used in further analyses.

### 2.4. Sleep Parameter

Sleep parameters were selected from the PSG database. The procedures used for PSG are described below. In-laboratory PSG was conducted using the ResMed Embla N7000 (ResMed, San Diego, CA, USA) and Embla MPR (Natus Medical, Pleasanton, CA, USA) systems. The PSG recorded various physiological signals, namely electroencephalography, electrooculography, electromyography (chin and leg), electrocardiography, nasal and oral airflow, snoring patterns, thoracic and abdominal impendence, sleeping position, and oxygen saturation. A licensed PSG technologist scored recordings using RemLogic software (version 3.41, Embla, Thornton, CO, USA), following the Americana Academy of Sleep Medicine Scoring Manual Version 2.4 [18]. All the scored results were reviewed by another technologist, and inconsistent scorings were identified and discussed further to achieve a consensus. We determined the distribution of each sleep stage, namely wake, rapid eye movement (REM), and non-REM (NREM), and we subsequently calculated the wake after sleep onset (WASO) accumulation time. OSA severity was classified by AHI into four levels: normal (AHI < 5 events/h), mild (5 ≤ AHI < 15 events/h), moderate (15 ≤ AHI < 30 events/h), and severe (AHI ≥ 30 events/h) [6]. For patients with AHI ≥ 15 events/h, OSA intervention was recommended [19]. We thus developed two types of risk screening models, one for the risk of moderate to severe OSA (AHI ≥ 15 vs. AHI < 15) and the other for the risk of severe OSA (AHI ≥ 30 vs. AHI < 30). 

### 2.5. Statistical Analysis

We employed Python (version 3.9.7) and an open-source statistics module, scikit-learn (version 0.21.2), to perform the statistical analyses. Patients were split into three groups according to OSA severity: normal-to-mild, moderate, and severe OSA groups. For continuous variables, the Shapiro–Wilk test was first used to examine the normality of their distribution. We employed nonparametric statistical approaches because the grouped data were nonnormally distributed. Subsequently, we used Levene’s test to examine the homogeneity of variance, followed by the Kruskal–Wallis test (homoscedastic) and Welch’s analysis of variance test (heteroscedastic). Regarding nominal variables, we used the chi-square test to compare intergroup differences. In addition, Pearson’s correlation was applied to determine the correlations between anthropometric features and sleep quality indices, namely AHI, ODI, snoring index, and arousal index. The level of statistical significance was set at *p* < 0.05.

### 2.6. Machine Learning Approaches

**Figure 1:**
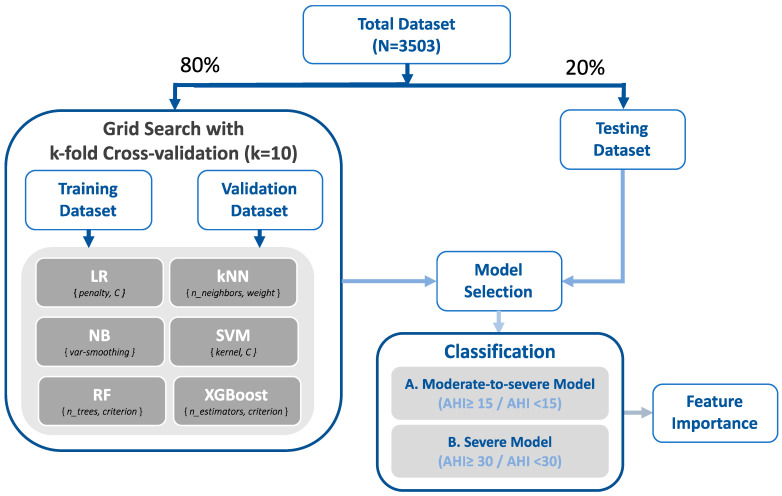
Training process with grid search cross-validation. Various machine learning models were trained using grid search cross-validation (k-fold: 10). The model demonstrating the highest accuracy in the validation stage was employed to predict the testing data, and the feature importance was investigated. Abbreviations: LR, logistic regression; C, regularization values; kNN, k-nearest neighbor; NB, naïve Bayes; var_smoothing, portion of the largest variance of all features; SVM, support vector machine; RF, random forest; n_trees, number of classifications and regression trees; XGBoost, extreme gradient boosting; n_estimators, number of gradient boosted trees; AHI, apnea–hypopnea index.

Six supervised machine learning models, namely, logistic regression (LR), k-nearest neighbors (kNN), naïve Bayes (NB), random forest (RF), support vector machine (SVM), and extreme gradient boosting (XGBoost), were employed to develop the two types of OSA risk screening models. Figure 1 illustrates the flowchart for the model establishment. Initially, the data were independently separated into two data sets (training and validation set and testing set) at a ratio of 80% and 20%. First, we applied grid search 10-fold cross-validation during the training and validation stage to determine the optimal classifier for each machine learning approach [20]. Specifically, we compared the accuracy by tuning (a) the inverse values of regularization (*C*, from 10^−5^ to 10^5^) for the LR models; (b) the k value (ranging from 2 to 5) and weight type (uniform or distance) for the kNN models; (c) the portion of the largest variance of all features for the NB models (var_smoothing, from 10^−9^ to 10^9^); (d) various kernel types (linear, polynomial, and radial basis function) and regularization values (*C*, between 10^−3^ and 10^3^) for the SVM models, (e) the criterion (Gini index or entropy) and the number of classification and regression trees (set as 250, 500, and 750) for the RF models with the bootstrap technique, and (f) the criterion (mean squared error (MSE), Friedman MSE, or squared error) and the number of estimators (set as 250, 500, and 750) for the XGBoost models. The performance matrix and area under the receiver operating characteristic curve (AUC) of each model were then determined. Thereafter, the machine learning approach with the highest accuracy was employed in the testing stage for further evaluation, and the Shapley values of the input variables for the employed models were calculated and visualized in a scatterplot to evaluate the contribution of each feature within the OSA risk screening models [21]. 

## 3. Results

### 3.1. Characterization of Enrolled Participants

We recruited a cohort of 3503 individuals for this retrospective study. Table 1 presents their anthropometric features grouped by OSA severity: normal-to-mild (AHI < 15), moderate (15 ≤ AHI < 30), and severe OSA groups (AHI ≥ 30). Regarding body profiles, the severe group demonstrated the highest mean values for BMI and neck and waist size, with the ratio of men being higher in the severe OSA group (1299/284, 82.06%) than in the normal-to-mild (348/603, 36.59%) and moderate OSA groups (677/292, 69.87%). For body composition parameters, the severe OSA group exhibited the highest mean values for fat mass and fat percentage (in the whole body, only limbs, and only trunk), visceral fat level, and basal metabolic rate, but the lowest mean values for physique rating and muscle mass as a percentage of whole-body mass (all *p* < 0.5). Similarly, for body water distribution, the severe OSA group had the highest mean values for TBW (40.18 kg ± 6.37 kg), ECW (16.5 kg ± 1.89 kg), and ICW (23.68 kg ± 4.63 kg), but the lowest mean values for body water percentage (48.59% ± 5.76%).

### 3.2. Sleep Parameters

The details of the sleep quality indices by sleep stage are presented in Table 2. The severe group exhibited the lowest mean values for sleep efficiency (72.5% ± 16.99%), total sleep time (264.96 min ± 62.5 min), and the mean (93.46% ± 2.58%) minimum values (77.14% ± 8.64%) of oxygen saturation measured through pulse oximetry (SpO2). Regarding sleep stage parameters, the patients with severe OSA demonstrated the highest percentage for the wake stage (22.06% ± 16.18%) and highest mean WASO time (73.98 min ± 53.48 min). Conversely, the severe OSA group had the lowest percentage for both the REM and NREM stages (REM: 10.1% ± 6.3%; NREM: 67.83% ± 13.84%). Regarding the sleep quality indices, the severe group had the highest mean values for AHI, ODI (≥3%), snoring index, and arousal index (all *p* < 0.05). By contrast, the normal-to-mild OSA group had the lowest mean value for all of these indices. 

### 3.3. Sleep Quality Index and Anthropometric Features

The correlations between the sleep quality index and anthropometric features are illustrated in Table 3. Regarding body profiles, BMI (ρ: 0.57), neck size (ρ: 0.59), and waist size (ρ: 0.61) had significant moderate correlations with AHI (all *p* < 0.05). For body composition parameters, fat mass, fat-free mass, and muscle mass (ρ: 0.4 to 0.48) in various body regions (i.e., whole body, only limbs, and only trunk) exhibited significant moderate correlations with AHI (all *p* < 0.05). Moreover, visceral fat level (ρ: 0.64) had a significant moderate to strong correlation with AHI (*p* < 0.05). In terms of body water distribution, AHI was positively correlated with TBW, ECW, and ICW (ρ: 0.43 to 0.58, *p* < 0.05), whereas AHI was negatively correlated with body water percentage (ρ: −0.24, *p* < 0.05). Moreover, the correlations of anthropometric features with other sleep quality indices, namely, ODI, snoring index, and arousal index, were similar to the correlations with AHI. 

### 3.4. Validation Performance of Machine Learning Approaches

The performance of the training and validation stage of each machine learning approach is summarized in Table 4. For the moderate to severe OSA model, the RF model exhibited the highest overall accuracy (LR: 81.55% ± 2.77%; kNN: 82.59% ± 2.05%; NB: 78.16% ± 3.29%; SVM: 83.51% ± 0.61%; RF: 85.19% ± 2.86%; XGBoost: 83.94% ± 2.52%). For the severe OSA model, similarly, the RF model outperformed the other models in accuracy (LR: 72.59% ± 0.99%; kNN: 70.52% ± 0.88%; NB: 72.66% ± 1.04%; SVM: 73.84% ± 0.04%; RF: 75.95% ± 2.24%; XGBoost: 73.91% ± 0.48%). In terms of AUC, similar to the accuracy results, the RF model demonstrated the highest values in both the moderate to severe (AUC: 90.41% ± 2.44%) and severe (83.24% ± 1.69%) OSA risk screening models. Because it had the strongest performance (highest accuracy and AUC), the RF models were adopted to predict the testing data sets and further explore their feature importance.

### 3.5. Accuracy Performance and Feature Importance

**Figure 2:**
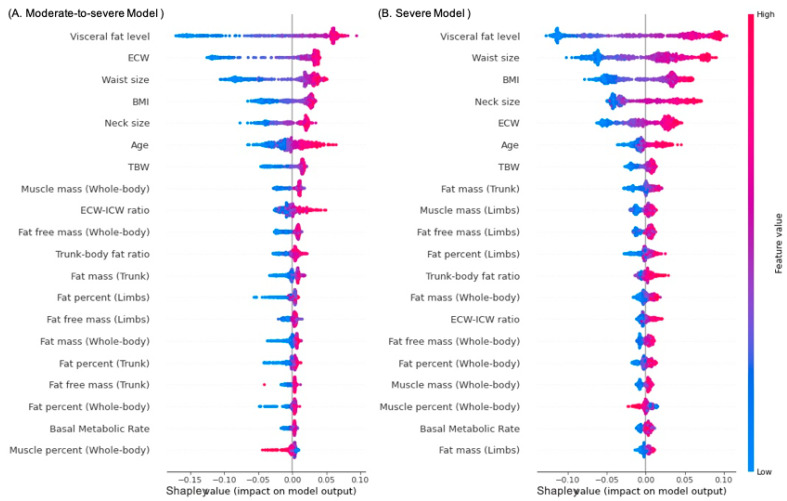
Density scatterplots representing Shapley values of input parameters for the RF models for screening moderate to severe and severe risks of OSA with the testing data set: (**A**) moderate-to-severe OSA model and (**B**) severe OSA model. Abbreviations: BMI, body mass index; TBW, total body water; ECW, extracellular water; ICW, intracellular water.

The model performance summary for the testing data set from the RF models is illustrated in Table 5. For the moderate-to-severe OSA RF model, the prediction accuracy was 84.74% and the AUC was 89.58%. For the severe OSA RF model, the prediction accuracy was 72.61% and the AUC was 80.07%. The feature importance in the RF models for the two risk types is presented in Figure 2. In the figure, the variables are arranged from top to bottom according to their Shapley values, with high and low values represented by red and blue dots, respectively. In both risk screening models (for moderate to severe and severe OSA), visceral fat level demonstrated the highest Shapley values, indicating the highest feature importance. Moreover, high visceral fat level (red dot) contributed to high OSA risk (high Shapley value). The ECW, neck and waist size, and BMI were alternately ranked from second to fifth highest in feature importance in both the risk screening models for moderate-to-severe OSA and severe OSA models.

### 3.6. Supplementary

We further developed OSA screening models for multiclass classification, including AHI < 15 (normal to mild OSA), 30 > AHI ≥ 15 (moderate), and AHI ≥ 30 (severe). The outcomes are presented in Supplementary Information. As shown in Table S1, the RF models exhibited the highest prediction accuracy (66.71%) and AUC (79.24%) in the training and validation stage. Subsequently, the RF models were used to predict the testing data sets because this approach outperformed other approaches; the outcomes are summarized in Table S2. The accuracy of multiclass prediction was 62.91%, and the AUC for this classification performance was 77.47%. Figure S1 illustrates feature importance in the RF models used for predicting the severity of OSA. Similar to the findings obtained using the moderate-to-severe and severe OSA models, the level of visceral fat exhibited the highest Shapley value in the RF models for multiclass prediction, which indicated its highest feature importance. Waist size, ECW, neck size, and BMI were sequentially ranked from second to fifth in terms of feature importance. 

## 4. Discussion

To develop robust models based on easily accessed parameters for OSA risk screening, we investigated the relationships of anthropometric features with PSG parameters by using a large sample from Taiwan (N = 3503). We conducted comparisons of the anthropometric features and PSG parameters of patients with different OSA severity. We also examined the correlations between sleep quality indices and anthropometric features, namely, body profiles and body composition parameters. Subsequently, various machine learning models based on anthropometric features were developed for screening the risk of moderate to severe OSA (AHI ≥ 15) and severe OSA (AHI ≥ 30). The models with the highest accuracy in the training and validation stage were used in the validation experiments; these models for both types of OSA severity exhibited high classification accuracy when using the testing data set. Moreover, we examined the feature importance of the adopted models in OSA severity screening. 

First, concerning model performance, the RF models using the bootstrap technique with optimal parameters derived from grid search cross-valuation demonstrated the highest accuracy and AUC in both types of OSA risk screening models. Similar to the results presented in Supplementary Information, the accuracy and AUC of the newly developed RF models were superior to the classification performance of other approaches. Although the literature does not provide evidence that RF outperforms other machine learning approaches, several plausible explanations may account for the present results. RF models, constructed per the theory of ensemble learning, may promote the accurate convergence of classification results (due in part to favorable antinoise ability) because this model architecture is more sensitive to relevant features and adept at disregarding the effects of irrelevant ones in comparison with other model architectures [22]. Moreover, the bootstrapping procedure and the number of decision trees in RF can be easily fine-tuned to avoid overfitting and maintain model stability [23]. Hence, the RF approach has been broadly employed for aiding medical diagnosis [24,25]. Compared with current machine learning approaches for screening OSA risk, the current models have some advantages. Specifically, our models are more suitable for application in clinical scenarios and demonstrate adequate accuracy. In related research, nocturnal oxygen saturation was adopted as a surrogate in OSA risk evaluation, and models integrating pulse oximetry data were developed [26]. However, variability in wearing situations, including incorrect probe placement, contact problems with the probe, and individuals’ body movements, may contribute to severe artefacts in the measurements [27]. Another study proposed machine learning models that integrated electrocardiogram data from wearable devices to screen for OSA risk; these models exhibited acceptable accuracy [28]. However, this type of screening method may not be suitable for patients with cardiopulmonary diseases because of the irregular and complex electrocardiogram signals of such patients. Moreover, some researchers have proposed machine learning models based on craniofacial feature images to predict OSA severity. Although craniofacial factors are significantly associated with OSA risk, the accuracy of those models was only 67% for classifying moderate to severe OSA [29]. This result may be attributed to poor precision caused by variations in captured craniofacial images and the fact that OSA pathology may not be entirely attributable to craniofacial factors. Prior studies have also proposed machine learning approaches for screening OSA risk by using different types of anthropometric features [30,31]; these methods exhibited relatively stable performance, and the interpretation of feature importance was straightforward. The literature thus suggests that machine learning models based on easily accessed anthropometric parameters may be practical for rapid screening of OSA severity in clinical scenarios. 

Regarding the importance of features used in the developed models, visceral fat level had the highest Shapley value, suggesting that it is a predominant factor for screening OSA risk. In terms of feature importance, BMI, neck size, and waist size followed visceral fat level. These outcomes were consistent with our statistical findings that these anthropometric parameters were correlated with AHI and ODI. These results can be partially attributed to body fat deposition, which can be estimated using visceral fat (internal organs), waist size (abdomen), neck size (upper airway), or BMI (whole body), being associated with obesity level and thus affecting AHI [32]. Several studies exploring fat accumulation in various body regions have suggested that body fat volume is associated with OSA risk [33,34]. Studies have also indicated that BMI and waist size are significantly associated with OSA [35] and suggested the feasibility of using BMI and neck size to predict OSA risk for men and women, respectively [36]. In addition, for visceral fat, a related study indicated that one of the clinical manifestations of OSA, nocturnal hypoxemia, was associated with increased inflammatory responses in adipose tissue and decreased insulin sensitivity [37]. These interplays can interfere with glucose uptake and stimulate hepatic gluconeogenesis, thereby increasing visceral fat accumulation. Biomechanically, the presence of visceral fat is associated with reduced thoracic capacity and lung volume [38], potentially increasing the workload of respiratory muscles and even resulting in more severe OSA. One study investigated the biomechanism of visceral adipocytes during oxygen starvation and observed that intermittent hypoxia may cause elevated oxidative stress and insulin resistance, aggravate the inflammatory effect, and trigger initial dysmetabolism [39].

Regarding the effect of body water distribution on OSA risk prediction, ECW was the second and fifth most important factor in the risk screening models for moderate to severe and severe OSA, respectively. These results are likely attributable to the associations between ECW and sleep apnea. Researchers have indicated that nocturnal Rostral fluid redistribution from the lower limbs was independent of body weight, and that it may increase the likelihood of upper airway narrowing, thereby contributing to OSA pathogenesis [40]. In another study, ECW was also associated with residual kidney function; the resulting fluid overload can elevate the mucosal water content in the upper airway and thereby aggravate OSA severity [41]. Studies comparing those with and without OSA have observed a higher percentage of ECW [42] as well higher mean values in the percentages of TBW and ECW [43] in those with OSA. Collectively, body water distribution, especially ECW, may serve as a practical indicator when screening the risk of OSA.

The present study has some strengths. First, using the models developed in this study, we can identify patients with moderate to severe and severe risk of OSA on the basis of easily acquired anthropometric parameters. The adequate classification performance of the models may help optimize PSG to increase the availability of medical resources by prioritizing high-risk patients for PSG and treatment. Second, although machine learning approaches have been described as a black box method, which means how input variables are combined to make predictions could not be determined, we evaluated the feature importance by calculating Shapley values; this may improve our understanding of correlations between OSA risk and various input parameters. Finally, considering the outcomes of the feature importance distribution, we may prevent OSA by reducing its effect. For instance, reducing the level of visceral fat should be considered first for reducing OSA risk. Similarly, because ECW was identified to be a key factor in leading OSA, performing exercises to reduce body fluid levels and, by extension, body water retention before sleep time may help reduce or eliminate the severity of OSA. 

The current study has some limitations that should be considered and addressed in future work. First, our models were population specific because we collected data exclusively from the populations in Taiwan. However, not only anthropometric features but also craniofacial factors have been reported to affect AHI and OSA severity [44]. Our models, therefore, may only be applied to specific ethnicities or populations with craniofacial features similar to the population in Taiwan. Second, our findings were based on PSG results; because PSG involves manual scoring, interscorer variability between PSG technologists may have affected our data quality. Although technicians from the same sleep center undergo regular scoring training, some degree of human variability is unavoidable [45]. Third, environmental factors, such as the first-night effect, may also have affected the quality of our data set [46]. More precisely, sleeping in a new environment may alter an individual’s sleep cycle and physiology, thereby causing inaccurate PSG outcomes. Although we excluded patients with low sleep efficiency, further works may consider using data from repeated PSG to prevent or reduce such bias [47]. Fourth, this retrospective study lacked information regarding lifestyle habits (tobacco and alcohol use) [48] or personal health status (menopausal status and comorbidities) [49,50]. Nevertheless, the association between OSA and these baseline details has been documented. Future research can consider obtaining these data by using questionnaires or retrospect patients’ disease-related parameters from personal medical history. Such additional data may be helpful for training more comprehensive models and increasing the accuracy of OSA risk screening.

## 5. Conclusions

To address the limitations of current screening tools for OSA severity, we developed novel models using easily accessed parameters. On the basis of anthropometric features obtained from 3503 patients in Taiwan, we developed various machine learning models to predict the risk of severe-to-moderate OSA and severe OSA. In the training and validation stage, the RF-based prediction models demonstrated the highest accuracy and AUC in both OSA severity risk categories among all the machine learning approaches. We, therefore, applied the RF models for testing data set prediction; the accuracy was 84.74% for the moderate to severe model and 72.61% for the severe model. Regarding feature importance, visceral fat level was the most critical feature in the OSA risk screening. Similarly, our statistical outcomes suggested that AHI and ODI significantly correlated with the anthropometric features related to obesity (i.e., BMI; neck size; waist size; visceral fat level; and the mass and percentage of fat in the whole body, only limbs, and only trunk). Our machine learning models may be employed to screen for OSA risk in populations with similar craniofacial features. 

## Figures and Tables

**Table 1:** Baseline characteristics and body composition parameters of participants grouped by OSA severity.

Categorical Variables	N-M Group (N = 951)	M Group (N = 969)	S Group (N = 1583)	Post Hoc
Age (years) ^a^	43.19 ± 13.85	49.48 ± 13.26	49.85 ± 13.04	N-M < M ** & S **
Sex (male to female ratio) ^b^	0.57 (348/603)	2.32 (677/292)	4.57 (1299/284)	N-M < M ** & S **; M < S **
BMI (kg/m^2^) ^c^	23.01 ± 3.33	26.55 ± 4.02	29.44 ± 4.83	N-M < M ** & S **; M < S **
Neck size (cm) ^a^	33.96 ± 3.12	37.36 ± 3.34	39.85 ± 3.48	N-M < M ** & S **; M < S **
Waist size (cm) ^c^	80.56 ± 9.49	91.35 ± 9.91	99.21 ± 11.03	N-M < M ** & S **; M < S **
Body composition data				
**Whole body**				
Fat mass (kg) ^c^	16.58 ± 6.87	21.14 ± 8.72	25.87 ± 10.94	N-M < M ** & S **; M < S **
Muscle mass (kg) ^c^	42.26 ± 8.69	50.13 ± 9.54	54.68 ± 9.17	N-M < M ** & S **; M < S **
Visceral fat level ^c^	6.77 ± 3.35	11.83 ± 3.76	14.89 ± 3.96	N-M < M ** & S **; M < S **
Bone mass (kg) ^c^	2.47 ± 0.44	2.84 ± 0.44	3.05 ± 0.42	N-M < M ** & S **; M < S **
Fat-free mass (kg) ^c^	44.73 ± 9.1	52.97 ± 9.96	57.74 ± 9.57	N-M < M ** & S **; M < S **
Fat percentage (%) ^a^	26.82 ± 8.63	28.13 ± 8.79	30.22 ± 8.79	N-M < S **; M < S **
Muscle percentage (%) ^a^	69.15 ± 8.32	68.02 ± 8.48	66.08 ± 8.45	N-M > S **; M > S **
Basal metabolic rate (kJ) ^c^	5415.96 ± 1009.17	6320.99 ± 1138.15	6919.51 ± 1144.22	N-M < M ** & S **; M < S **
Physique rating ^c^	36.07 ± 9.89	29.5 ± 9.36	25.44 ± 8.7	N-M > M ** & S **; M > S **
**Limbs**				
Fat mass (kg) ^c^	7.46 ± 2.91	8.95 ± 3.81	10.97 ± 5.13	N-M < M ** & S **; M < S **
Fat-free mass (kg) ^c^	20.75 ± 4.96	25.1 ± 5.83	28.11 ± 5.92	N-M < M ** & S **; M < S **
Muscle mass (kg) ^c^	19.54 ± 4.67	23.65 ± 5.52	26.5 ± 5.63	N-M < M ** & S **; M < S **
Fat percentage (%) ^c^	26.58 ± 8.49	26.13 ± 8.52	27.42 ± 8.16	N-M * & M ** < S
**Trunk**				
Fat mass (kg) ^c^	9.12 ± 4.07	12.19 ± 5.01	14.9 ± 6.04	N-M < M ** & S **; M < S **
Fat-free mass (kg) ^c^	23.98 ± 4.44	27.87 ± 4.5	29.62 ± 4.42	N-M < M ** & S **; M < S **
Muscle mass (kg) ^c^	22.72 ± 4.29	26.48 ± 4.38	28.18 ± 4.28	N-M < M ** & S **; M < S **
Fat percentage (%) ^a^	27.01 ± 9.09	29.85 ± 9.27	32.72 ± 9.63	N-M < M ** & S **; M < S **
Trunk to whole-body fat ratio (%) ^c^	54.24 ± 5.11	57.61 ± 3.36	57.85 ± 3.21	N-M < M ** & S **
**Body water**				
TBW (kg) ^a^	31.33 ± 6.09	36.84 ± 6.39	40.18 ± 6.37	N-M < M ** & S **; M < S **
ECW (kg) ^c^	12.89 ± 2.06	15.22 ± 1.93	16.5 ± 1.89	N-M < M ** & S **; M < S **
ICW (kg) ^c^	18.44 ± 4.19	21.62 ± 4.61	23.68 ± 4.63	N-M < M ** & S **; M < S **
Body water percentage (%) ^c^	51.25 ± 5.41	50.03 ± 5.35	48.59 ± 5.76	N-M > M ** & S **; M > S **
ECW to ICW ratio (%) ^c^	71.14 ± 7.88	71.98 ± 8.66	71.03 ± 8.14	M > S *

Abbreviations: N-M group, normal-to-mild OSA group; M group, moderate OSA group; S group, severe OSA group; BMI, body mass index; TBW, total body water; ECW, extracellular water; and ICW, intracellular water. Data are expressed in terms of mean ± standard deviation values. Significant difference was derived from ^a^ Kruskal–Wallis test, ^b^ chi-square test, and ^c^ Welch’s analysis of variance test. * *p* < 0.05 and ** *p* < 0.01.

**Table 2:** Polysomnography parameters of participants grouped by OSA severity.

Categorical Variables	N-M Group (N = 951)	M Group (N = 969)	S Group (N = 1583)	Post Hoc
Sleep efficiency (%) ^a^	75.27 ± 16.16	74.64 ± 16.5	72.5 ± 16.99	N-M > S **; M > S **
Mean SpO_2_ (%) ^b^	96.35 ± 1.44	95.28 ± 1.51	93.46 ± 2.58	N-M > M ** & S **; M > S **
Minimum SpO_2_ (%) ^b^	90.06 ± 4.7	84.48 ± 6.04	77.14 ± 8.64	N-M > M ** & S **; M > S **
WASO (min) ^b^	54.1 ± 46.93	62.91 ± 50.0	73.98 ± 53.48	N-M < M ** & S **; M < S **
Total sleep time (min) ^b^	276.4 ± 60.66	273.7 ± 60.72	264.96 ± 62.5	N-M > S **; M > S **
**Sleep stage (% of SPT)**				
Wake ^b^	16.64 ± 14.7	18.96 ± 15.42	22.06 ± 16.18	N-M < M ** & S **; M < S **
NREM ^b^	71.26 ± 12.59	69.33 ± 12.87	67.83 ± 13.84	N-M > M ** & S **; M > S *
REM ^a^	12.03 ± 6.9	11.68 ± 6.47	10.1 ± 6.3	N-M > S **; M > S **
**Sleep quality index (events/h)**				
AHI ^b^	7.81 ± 4.3	21.68 ± 4.24	56.5 ± 21.18	N-M < M ** & S **; M < S **
ODI ^b^	4.02 ± 3.65	14.81 ± 6.46	48.84 ± 23.44	N-M < M ** & S **; M < S **
Snoring index ^b^	99.73 ± 158.35	216.41 ± 214.94	315.69 ± 220.36	N-M < M ** & S **; M < S **
Arousal index ^b^	13.81 ± 7.79	18.27 ± 9.28	32.67 ± 18.13	N-M < M ** & S **; M < S **

Abbreviations: N-M group, normal-to-mild OSA group; M group, moderate OSA group; S group, severe OSA group; SpO_2_, oxygen saturation measured using pulse oximetry; WASO, wake after sleep onset; SPT, sleep period time; NREM, nonrapid eye movement; REM, rapid eye movement; AHI, apnea–hypopnea index; and ODI, oxygen desaturation index (≥3%). Data are expressed in terms of mean ± standard deviation values. Significant difference was derived from the ^a^ Kruskal–Wallis test and ^b^ Welch’s analysis of variance test. * *p* < 0.05 and ** *p* < 0.01.

**Table 3:** Pearson’s correlation coefficients for polysomnography parameters and body composition parameters.

Categorical Variable	Sleep Quality Index (events/h)			
	AHI	ODI	Snoring Index	Arousal Index
Age (years)	0.11 **	0.08 **	0.06 **	0.09 **
Sex (male/female)	0.33 **	0.30 **	0.17 **	0.21 **
BMI (kg/m^2^)	0.57 **	0.59 **	0.4 **	0.3 **
Neck size (cm)	0.59 **	0.58 **	0.38 **	0.36 **
Waist size (cm)	0.61 **	0.61 **	0.41 **	0.35 **
**Body composition**				
**Whole body**				
Fat mass (kg)	0.45 **	0.47 **	0.32 **	0.23 **
Muscle mass (kg)	0.46 **	0.45 **	0.29 **	0.28 **
Visceral fat level	0.64 **	0.62 **	0.41 **	0.36 **
Bone mass (kg)	0.47 **	0.47 **	0.31 **	0.28 **
Fat-free mass (kg)	0.47 **	0.45 **	0.29 **	0.28 **
Fat percentage (%)	0.22 **	0.24 **	0.18 **	0.08 **
Muscle percentage (%)	−0.21 **	−0.23 **	−0.17 **	−0.08 **
Basal metabolic rate (kJ)	0.49 **	0.49 **	0.31 **	0.28 **
Physique rating	−0.41 **	−0.42 **	−0.3 **	−0.2 **
**Limbs**				
Fat mass (kg)	0.42 **	0.44 **	0.3 **	0.22 **
Fat-free mass (kg)	0.48 **	0.48 **	0.3 **	0.28 **
Muscle mass (kg)	0.48 **	0.48 **	0.3 **	0.28 **
Fat percentage (%)	0.11 **	0.14 **	0.12 **	0.03
**Trunk**				
Fat mass (kg)	0.46 **	0.47 **	0.32 **	0.23 **
Fat-free mass (kg)	0.4 **	0.38 **	0.24 **	0.25 **
Muscle mass (kg)	0.4 **	0.38 **	0.24 **	0.25 **
Fat percentage (%)	0.29 **	0.31 **	0.22 **	0.13 **
Trunk to body fat ratio (%)	0.22 **	0.19 **	0.13 **	0.12 **
**Body water**				
TBW (kg)	0.49 **	0.48 **	0.32 **	0.29 **
ECW (kg)	0.58 **	0.56 **	0.38 **	0.34 **
ICW (kg)	0.43 **	0.43 **	0.28 **	0.26 **
Body water percent (%)	−0.24 **	−0.26 **	−0.17 **	−0.1 **
ECW to ICW ratio (%)	−0.05 *	−0.05 **	−0.03 *	−0.04 *

Abbreviations: AHI, apnea–hypopnea index; ODI, oxygen desaturation index (≥3%); BMI, body mass index; TBW, total body water; ECW, extracellular water; ICW, intracellular water. Data are expressed as coefficients. * *p* < 0.05; ** *p* < 0.01.

**Table 4:** Accuracy of the models for screening moderate to severe and severe OSA when using grid search cross-validation with the training data set.

Categorical Variables	LR	kNN	NB	SVM	RF	XGBoost
**Moderate to severe OSA model**	AHI ≥ 15 (N = 2039)/AHI < 15 (N = 763)					
Precision	91.4 ± 2.22	86.46 ± 1.77	90.3 ± 2.84	83.1 ± 0.79	86.72 ± 1.93	86.75 ± 1.01
Recall	82.44 ± 2.91	90.24 ± 1.93	78.47 ± 2.96	97.11 ± 1.45	94.07 ± 1.99	91.96 ± 2.71
Accuracy	81.55 ± 2.77	82.59 ± 2.05	78.16 ± 3.29	83.51 ± 0.61	85.19 ± 2.86	83.94 ± 2.52
F1 score	86.66 ± 2.07	88.29 ± 1.38	83.94 ± 2.44	89.55 ± 0.43	90.24 ± 1.88	89.27 ± 1.78
AUC	90.03 ± 2.67	83.43 ± 3.64	87.41 ± 3.27	89.86 ± 2.02	90.41 ± 2.44	88.64 ± 1.74
**Severe OSA model**	AHI ≥ 30 (N = 1268)/AHI < 30 (N = 1534)					
Precision	68.29 ± 1.22	67.08 ± 0.84	68.9 ± 1.07	71.32 ± 0.16	70.67 ± 2.86	70.3 ± 0.7
Recall	73.66 ± 1.26	68.45 ± 1.91	72.16 ± 1.67	70.58 ± 0.24	76.57 ± 3.39	73.34 ± 0.83
Accuracy	72.59 ± 0.99	70.52 ± 0.88	72.66 ± 1.04	73.84 ± 0.04	75.95 ± 2.24	73.91 ± 0.48
F1 score	70.86 ± 1.02	67.75 ± 1.19	70.49 ± 1.25	70.95 ± 0.04	73.44 ± 2.28	71.79 ± 0.46
AUC	80.87 ± 0.36	75.97 ± 0.64	80.8 ± 0.5	83.13 ± 0.17	83.24 ± 1.69	81.59 ± 0.71

Abbreviations: AHI, apnea–hypopnea index; LR, logistic regression; kNN, k-nearest neighbors; NB, naïve Bayes; SVM, support vector machine; RF, random forest; XGBoost, extreme gradient boosting; AUC, area under the curve. Data are expressed as the mean and standard deviation

**Table 5:** Classification of the results of the random forest model for screening moderate to severe and severe risks of OSA by using the testing data set.

Categorical Variables	Moderate-to-Severe OSA Model	Severe OSA Model
	AHI ≥ 15 (N = 513); AHI < 15 (N = 188)	AHI ≥30 (N = 315); AHI < 30 (N = 386)
Precision %	85.74	67.72
Recall %	94.93	74.6
Accuracy %	84.74	72.61
F1 Score %	90.1	71.0
AUC, % (95% CI)	89.58 (87.44–92.01)	80.07 (77.05–82.80)

Abbreviations: AHI, apnea–hypopnea index; AUC, area under the curve; CI, confidence interval.

## Data Availability

All the data of this study were collected at the Sleep Center of Taipei Medical University–Shuang Ho Hospital (New Taipei City, Taiwan) between May 2019 and December 2021. Because our data set contains personal information, it is not available in the Supplementary File. For access to the data set or relevant documents, please contact the corresponding author.

## Supplementary Materials

The following supporting information can be downloaded at: https://www.mdpi.com/article/10.3390/s22228630/s1, Figure S1: Density scatterplots showing the Shapley values of the input parameters used in the RF models for assessing OSA severity using the testing data set; Table S1: Classification of the results of the random forest model used to assess the severity of OSA using the testing data set; Table S2: Classification of the results of the random forest model used to assess the risk of OSA using the testing data set.
