# Supplementary material for: Screening for Obstructive Sleep Apnea Risk by Using Machine Learning Approaches and Anthropometric Features
Source: Sensors (Basel). 2022 Nov 9;22(22):8630. doi: 10.3390/s22228630 (PMC9694257; doi:10.3390/s22228630)
Supplement: Supplementary file 1 [file sensors-22-08630-s001.zip › sensors-1962774-supplementary.pdf]

sensors

MDPI

Supplementary Information

Table S1. Classification of the results of the random forest model used to assess the severity of OSA using the testing data set.

Categorical variables	LR	kNN	NB	SVM	RF	XGBoost
**OSA severity classification**	AHI ≥ 30 (N = 1,268) / 30 > AHI ≥ 15 (N = 771) / AHI < 15 (N = 763)					
Precision	53.68 ± 9.29	56.3 ± 2.54	56.83 ± 2.58	57.82 ± 1.8	61.54 ± 3.72	59.85 ± 3.11
Recall	56.98 ± 1.51	56.14 ± 2.7	57.39 ± 2.82	58.32 ± 0.97	60.71 ± 2.75	59.94 ± 2.57
Accuracy	62.96 ± 1.5	58.74 ± 2.46	57.96 ± 2.83	62.92 ± 0.89	66.71 ± 2.32	64.06 ± 2.24
F1 score	49.15 ± 1.51	56.08 ± 2.62	56.69 ± 2.76	56.31 ± 1.79	61.12 ± 2.71	59.89 ± 2.8
AUC	78.49 ± 1.52	73.52 ± 2.18	76.62 ± 2.36	78.82 ± 0.08	79.24 ± 1.87	79.17 ± 1.39

Abbreviations: AHI, apnea–hypopnea index; LR, logistic regression; kNN, k-nearest neighbors; NB, naïve Bayes; SVM, support vector machine; RF, random forest; XGBoost, extreme gradient boosting; and AUC, area under the curve.

Data are expressed as the mean and standard deviation.

Table S2. Classification of the results of the random forest model used to assess the risk of OSA using the testing data set.

Categorical variables	OSA severity model
	AHI ≥ 30 (N = 315) / 30 > AHI ≥ 15 (N = 198) / AHI < 15 (N = 188)
Precision, %	57.26
Recall, %	57.36
Accuracy, %	62.91
F1 score, %	57.99
AUC, % (95% CI)	77.47 (73.19–81.52)

Abbreviations: AHI, apnea–hypopnea index; AUC, area under the curve; and CI, confidence interval.

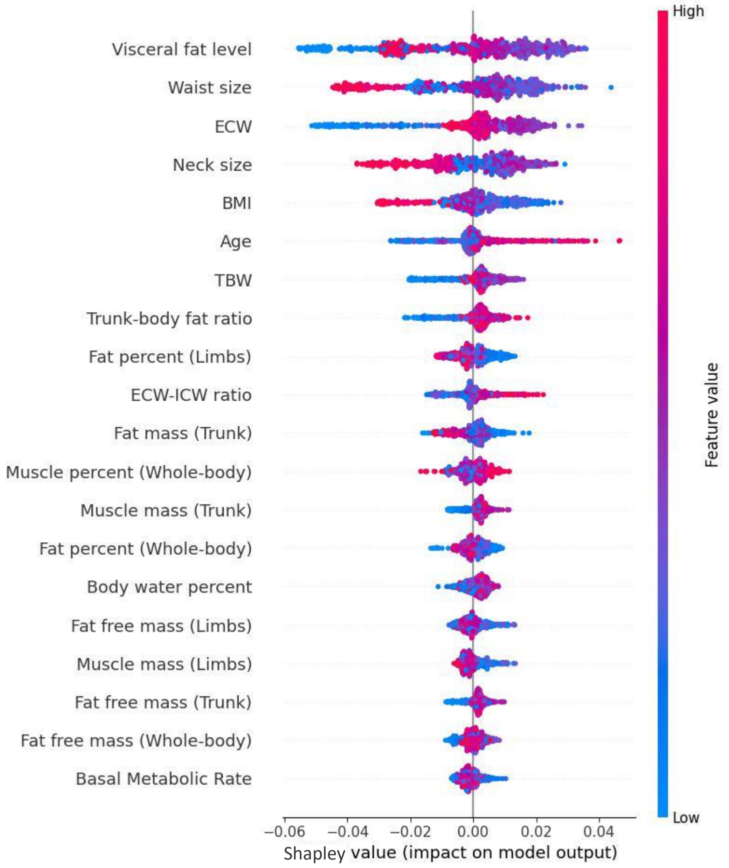

Figure S1. Density scatterplots showing the Shapley values of the input parameters used in the RF models for assessing OSA severity (normal to mild [AHI < 15], moderate [30 > AHI ≥ 15], and severe [AHI ≥ 30]) using the testing data set.
